# Rh(I) Complexes in Catalysis: A Five-Year Trend

**DOI:** 10.3390/molecules26092553

**Published:** 2021-04-27

**Authors:** Serenella Medici, Massimiliano Peana, Alessio Pelucelli, Maria Antonietta Zoroddu

**Affiliations:** Department of Chemistry and Pharmacy, University of Sassari, Vienna 2, 07100 Sassari, Italy; alessiopelucelli@gmail.com (A.P.); zoroddu@uniss.it (M.A.Z.)

**Keywords:** rhodium, catalysis, Rh(I) complexes

## Abstract

Rhodium is one of the most used metals in catalysis both in laboratory reactions and industrial processes. Despite the extensive exploration on “classical” ligands carried out during the past decades in the field of rhodium-catalyzed reactions, such as phosphines, and other common types of ligands including N-heterocyclic carbenes, ferrocenes, cyclopentadienyl anion and pentamethylcyclopentadienyl derivatives, etc., there is still lively research activity on this topic, with considerable efforts being made toward the synthesis of new preformed rhodium catalysts that can be both efficient and selective. Although the “golden age” of homogeneous catalysis might seem over, there is still plenty of room for improvement, especially from the point of view of a more sustainable chemistry. In this review, temporally restricted to the analysis of literature during the past five years (2015–2020), the latest findings and trends in the synthesis and applications of Rh(I) complexes to catalysis will be presented. From the analysis of the most recent literature, it seems clear that rhodium-catalyzed processes still represent a stimulating challenge for the metalloorganic chemist that is far from being over.

## 1. Introduction

Rhodium is one of the most used metals in catalysis, since it favours a wide range of chemical transformations that are fundamental for the organic synthesis, both in laboratory reactions or industrial processes.

Wilkinson’s chloridotris(triphenylphosphine)rhodium(I), [RhCl(PPh_3_)_3_] [1] was the first complex introduced in homogeneous catalysis for the hydrogenation of olefins (followed by several other hydrofunctionalization reactions, such as alkene hydroformylation, hydroboration, and hydrosilylation) and paved the way for the future development of chirally assisted processes. Since its introduction, an impressive number of rhodium complexes have been prepared and applied in catalysis [2,3] with more or less success. A vast gamut of ligands and donor atoms have been scrutinized in order to find the best catalyst for a specific reaction (or even for a specific substrate) in terms of activity and selectivity. However, although it is possible to identify a general trend in the reactivity of their metal complexes, no metal nor ligand are universal in catalysis, and every researcher devoted to this field of investigation knows that finding the perfect match of metal centre, ligand, substrate, and reaction conditions is often a matter of sheer luck.

**Chart 1:**
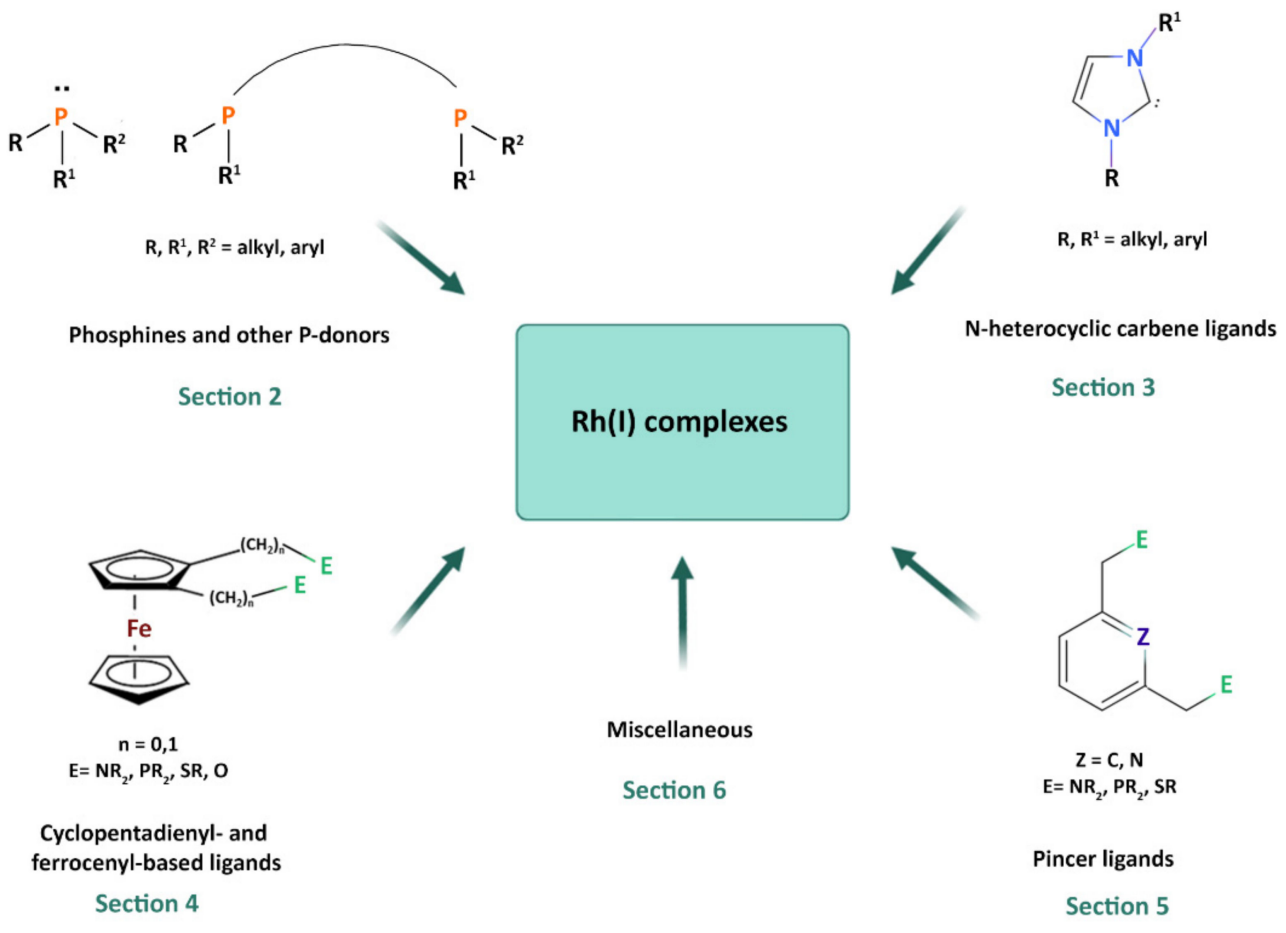
Graphical scheme for ligands commonly used in Rh(I) complexes for catalytic processes discussed in this work.

Classical ligands (Chart 1) with wide applications are phosphines [4], phosphites [5] and other P-donor species, aliphatic and aromatic nitrogen compounds [6], sulfur-ligands [7], and metalating or cyclometalating species [8], together with their chiral counterparts for the enantioselective catalysis [9,10,11,12,13]. Atropisomeric structures such as those represented by binaphthyl and, less commonly, biphenyl backbones were extensively exploited in the design of effective ligands employed in asymmetric reactions (e.g., BINAP, BIPHEP, SEGPHOS, and their derivatives) [14,15].

**Chart 2:**
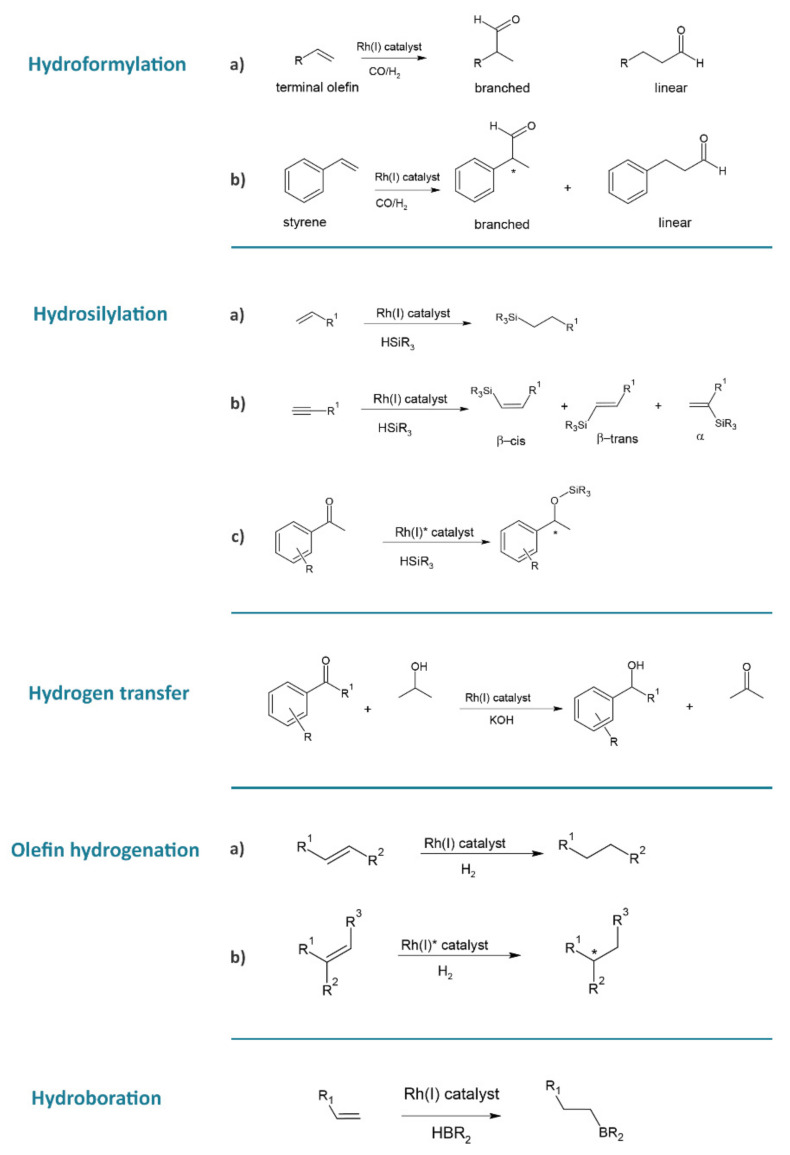
General reaction schemes for most common catalytic processes.

In situ preparation of the catalysts by addition of the ligand to a proper precursor, such as [Rh(COD)Cl]_2_, is a fast and easy way to test them in almost all the catalytic processes (Chart 2); still, the use of preformed Rh catalysts, precatalysts, or intermediates can give the opportunity to better study the reaction mechanisms and at the same time experience the benefits of improved selectivity and activity of the isolated catalytic species, together with reduced metal loading and ligand-to-metal ratios.

The exigence of decreasing the environmental and economic impact of metal-catalyzed industrial processes led to the search of alternatives to classic homogeneous catalysis carried out in organic solvents. Consolidating trends are the incorporation of Rh complexes in nanomaterials, use of biphasic media, and immobilization onto solid supports, just to quote some [16]. In this review, temporally restricted to the analysis of literature during the past five years, the latest findings and trends in the design and applications of Rh(I) complexes to catalysis will be presented.

## 2. Phosphines and Other P-Donors

**Chart 3:**
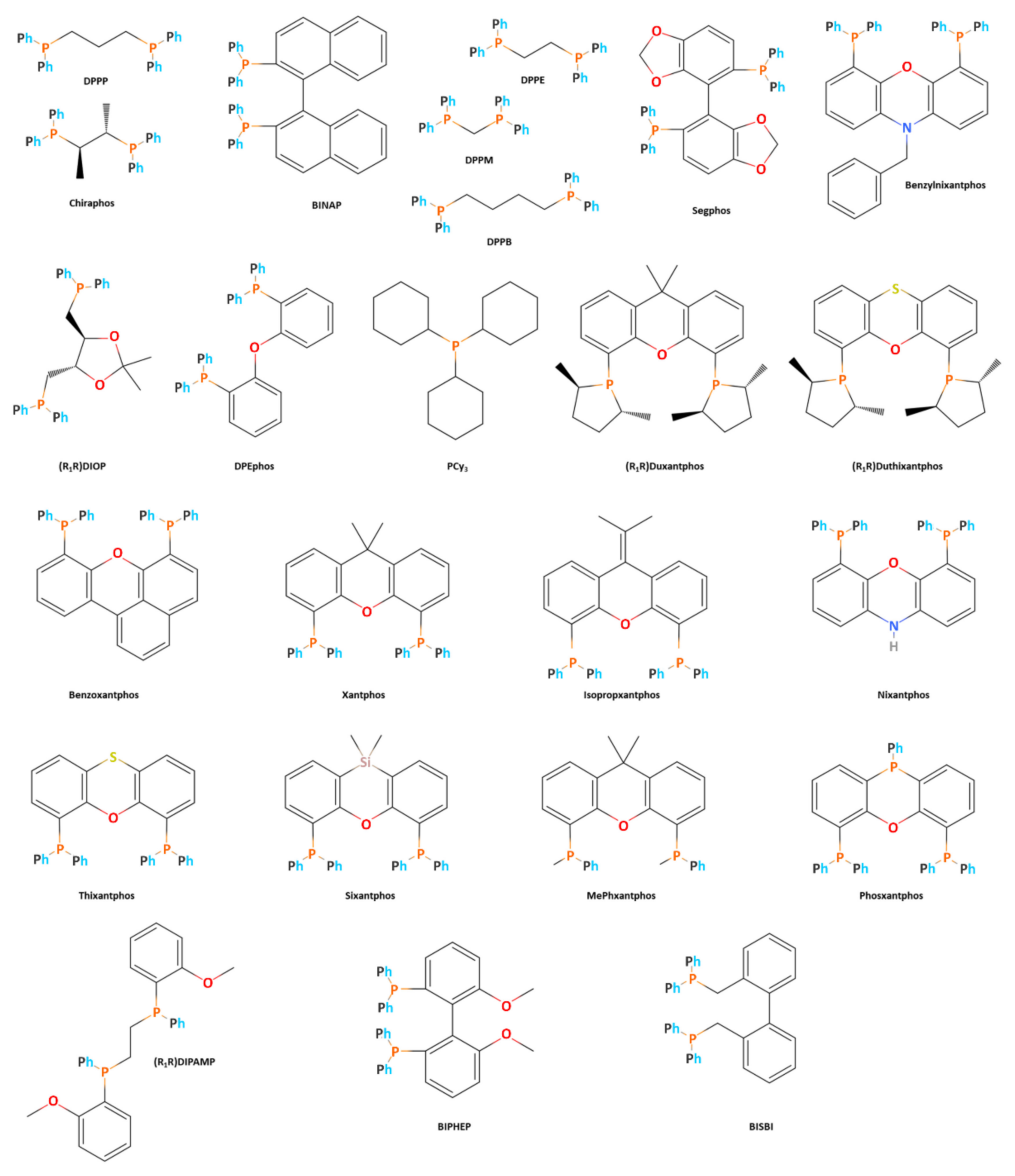
Molecular structures of the most used phosphines in catalysis.

Phosphorus–donor compounds have been extensively used in the preparation of transition metal complexes for homogeneous catalysis [17], since both the steric and electronic properties of organophosphorus species, and consequently those of the relative complexes, can be easily tuned by proper substitution. Phosphines are one of the most exploited classes of ligands for the synthesis of active metal-catalysts, including rhodium-based species. Both mono- and diphosphines found broad applications, especially those with simple design and relatively low cost, like PPh_3_, DPPE, DPPP, and DPPB [18,19], but chiral ones as well, like Chiraphos or atropisomeric BINAP and SEGPHOS, for asymmetric synthesis (Chart 3).

Phosphine groups are present on “pincer” ligands of the PCP or PNP type [20] (also in their chiral versions) [21] or in other mixed species, often associated to sulfur or oxygen and nitrogen donors, to afford hemilabile ligands. In fact, phosphorus is a “soft” donor that strongly binds to “soft” metal centres such as Rh(I) ions, while nitrogen and oxygen are relatively “hard” donors and therefore rather labile with this metal ion. Such lability can generate a coordination vacancy on the metal during the catalytic process while providing a higher coordination number and enhanced stability of the complex, both as the catalyst precursor or the actual catalyst in its resting state. Phosphites and phosponites have been extensively employed in catalysis as well. Compared to phosphines, they are able to better stabilize low oxidation state transition metals, because the presence of oxygen atoms, being more electronegative than their carbon counterparts, lowers the energy of their σ* orbitals; they are also poor σ-donors and strong π-acceptors. Such properties make them useful for the application in catalysis. The synthesis of new P-containing ligands during the past few years does not offer appreciable breakthroughs, since the possible structures for this class of molecules have been extensively explored in the previous decades, but still some advances have been made with the preparation of a few novel ligands. In the rest of the cases, known P-compounds were slightly modified or simply used as such in the preparation of new coordinated species in association with other co-ligands to give fresh Rh(I) complexes for catalysis applications.

### 2.1. New P-Ligands

Novel ligands include a series of PNP species [22] obtained via the modification of a previously reported procedure, which gave N-substituted iminodiphosphines with different groups (cyclohexyl, iso-propyl, pentyl, phenyl, chlorophenyl, and methoxyphenyl) in order to check their influence on the yield of the catalytic process by tuning the steric and/or electronic properties of the complexes. These kinds of ligands have been widely applied to the oligomerization of ethylene, but in this case their rhodium complexes were studied in the oxidation of styrene using tert-butyl hydroperoxide (TBHP) as the oxidant, with good activities and a higher propensity to give the benzaldehyde product respect to styrene oxide.

**Figure 1:**
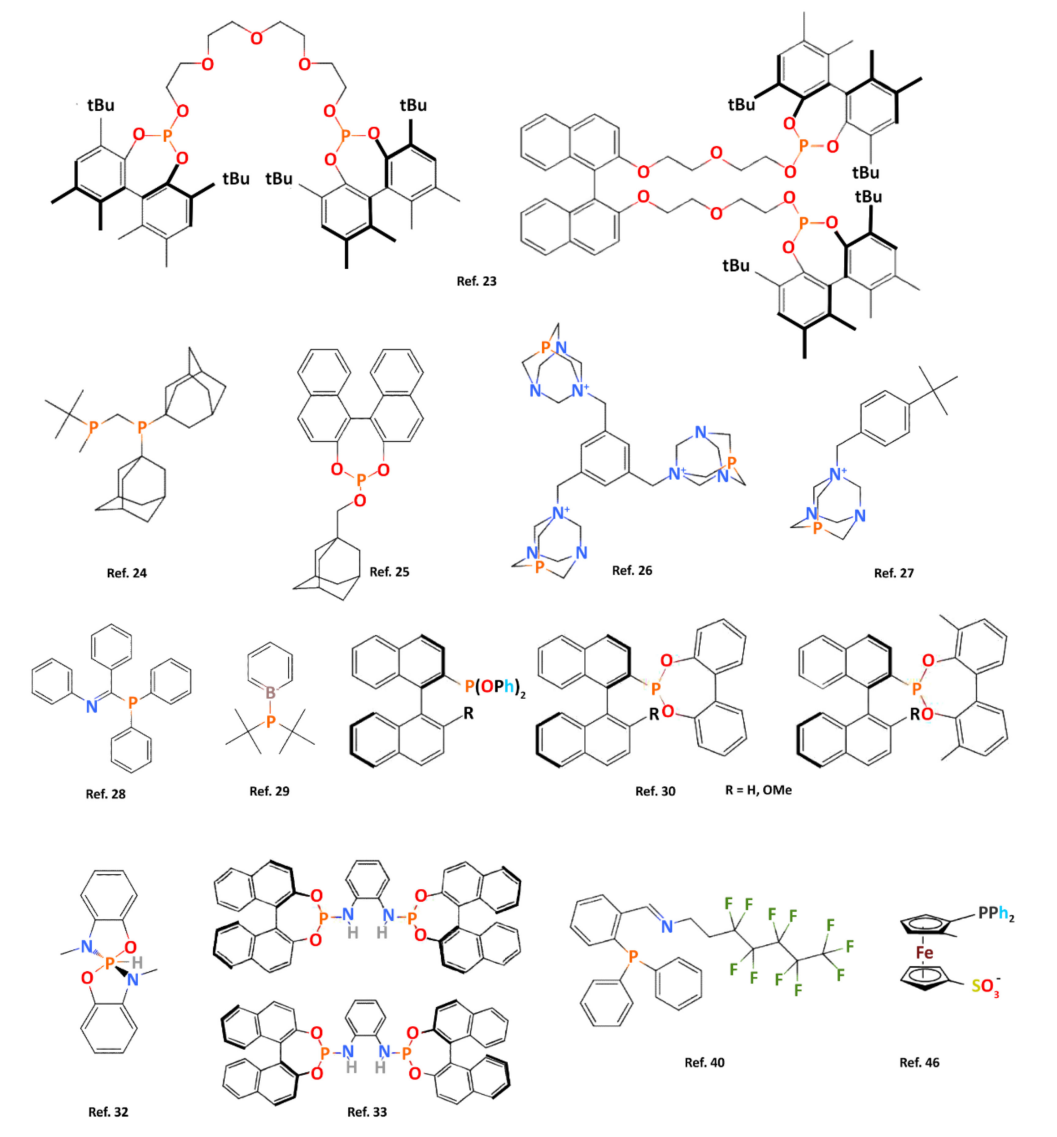
Molecular structures of some P-ligands in recent literature.

Two new chiral bisphosphonate species based on atropisomeric moieties have been prepared in a supermolecular arrangement [23] (Figure 1), which were then distally regulated by BArF_4_ salts in their coordination to a Rh(I) centre. In particular, these structures consisted of two phosphonate derivatives of a chiral biphenyl system linked via a polyether chain that was further expanded to incorporate a second axially chiral unit in the form of a BINOL spacer. The diastereomers thus obtained were tested in the asymmetric hydroformylation of heterocyclic olefins with good results in terms of both regio- and enantioselectivity. A peculiar three-hindered quadrant bisphosphine [24] (Figure 1) was synthesized to improve the results of an analogous ligand called trichickenfootphos (TCFP) that showed high catalytic activity in the rhodium-catalyzed asymmetric hydrogenation of functionalized alkenes. The problem with TCFP was its occurrence as an air sensitive oil that strongly limited its use. The new chiral ligand, (R)-di-1-adamantylphosphino-(tert-butylmethylphosphino)methane, named (R)-BulkyP* due to the presence of the massive di-1-adamantylphosphino group, is an air stable crystalline solid with an extra shield on two of the four quadrants, which increased both the efficiency and enenatioselectivity of the relative Rh(I) complex as the catalyst in the asymmetric hydrogenation of β-dehydroamino acid derivatives and other functionalized olefins, with yields higher than 95% and ees up to 99%.

The bulky adamantane group has also been exploited to prepare a novel sterically crowded monodentate phosphite ligand whose [Rh(COD)L_2_]BF_4_ complex was tested with good results in the asymmetric hydrogenation of dimethylitaconate, α- and β-enamides, and 2-methylindole. As is often the case, the structure of the molecule is constituted by a binaphthyl skeleton on which the phosphite moiety has been appended [25] (Figure 1). 

The adamantane backbone is a flexible structure that can be variously functionalized and substituted. PTA (1,3,5-triaza-7-phosphaadamantane) is the basis of mono-, di-, and trivalent phosphorus-based ligands, which were synthesized by the lower-rim benzylation of this compound. The corresponding mono-, di-, and trimetallic RhL(COD)Cl or RhL(CO)_2_Cl systems were investigated in the hydroformylation of 1-octene, to understand, among all, whether the increased number of metal centres could influence activity and selectivity [26] (Figure 1). COD complexes were more active than the analogous CO species, reaching TOFs higher than 200 h^−1^, and more selective towards the aldehyde product. On the other hand, CO complexes were more regioselective for linear aldehydes. Nuclearity influenced the catalytic rate, increasing in the order 1 < 2 < 3, but it had no effect on the regio- and chemoselectivity.

One of the most recent trends in the preparation of new organometallic complexes suitable for catalytic applications is the self-assembly approach of supramolecular structures by means of host–guest interactions. Such strategies have been explored for different processes, from metal-directed self-assembly to acid–base interactions. For instance, inclusion of a water-soluble phosphine ligand inside the β-cyclodextrin (β-CD) cavity by its NH_2_-containing face can afford a rigid chelating bidentate ligand suitable for complexation with transition metals, including rhodium. In this context, water-soluble ligands derived from a lower rim PTA derivative (Figure 1), that is a tailored N-quaternization of the cage-like aminophosphine PTA, and their interactions with β-CDs were studied [27]. The corresponding Rh complex was stabilized by inclusion in a mono-amino β-CD (β-CDNH_2_), and this supramolecular structure was assessed in the hydrogenation of unsaturated and allylic alcohols carried out in water.

### 2.2. Already Reported P-Ligands

Several new rhodium complexes with previously reported P-based ligands have been synthesized for catalytic applications, with interesting results.

Hemilabile P,N species based on acyclic and aprotic phosphaamidines and phosphaguanidines [28] (Figure 1) have been prepared according to literature procedures with phenyl or isopropyl groups in order to tune the lability of the N-donor. Rh (I) complexes as precatalysts for the homogeneous hydrogenation of CO_2_ in the production of formic acid have been prepared and studied with good results.

**Scheme 1:**
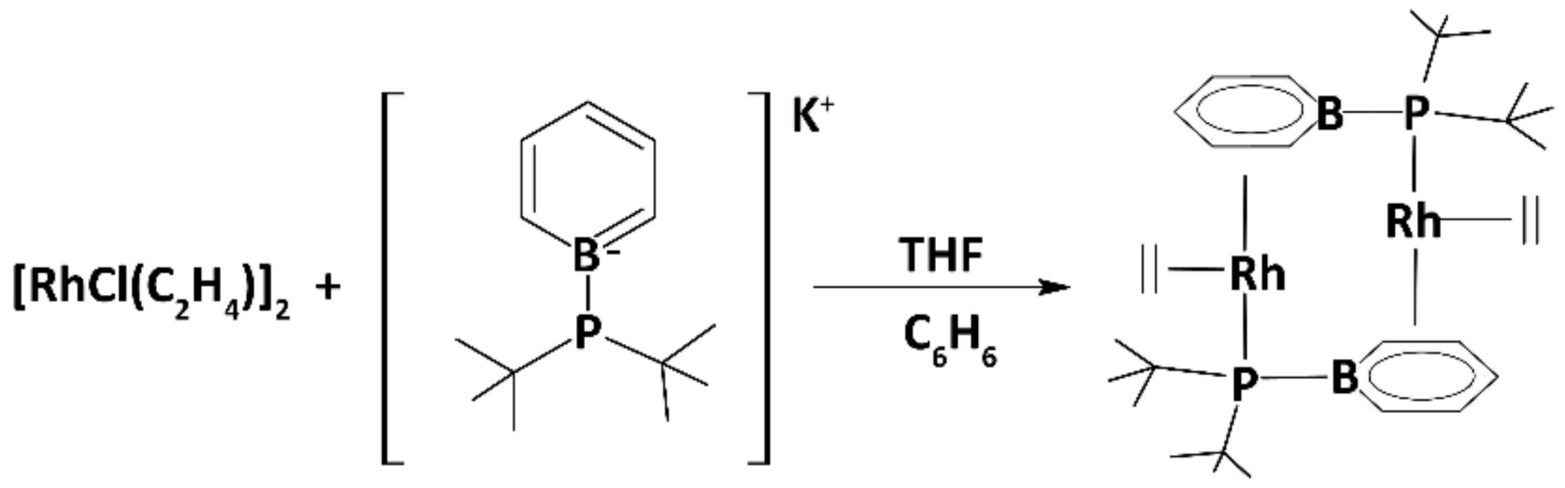
Reaction scheme for the synthesis of [(C_2_H_4_)Rh(DTBB)]_2._

Boron-heterocycles, either neutral (borabenzene adducts) or anionic (boratabenzenes), are recent compounds with an electronic structure analogous to Cp (cyclopentadienyl anion) that have been used to prepare transition metal complexes with interesting applications, such as the Rh-catalyzed C-H bond activation. Di-tert-butylphosphido-boratabenzene (DTBB) was developed to behave like a bulky and anionic phosphine, where the aromatic ring is able to coordinate the metal centre either in a η^3^ or η^6^ fashion, depending on the metal. In all respects, this is a hemilabile ligand, and thus very interesting for catalytic applications [29] (Figure 1). The relative Rh(I) complex was prepared from [Rh(C_2_H_4_)_2_Cl]_2_ (Scheme 1) as a bimetallic species with two bridging DTBB ligands where the aromatic ring was η^6^-coordinated to the metal. The catalyst was tested in the hydrogenation of unsaturated hydrocarbons at room conditions, with an activity comparable to that recorded for Wilkinson’s catalyst in similar conditions. 

Phosphonites are another class of organophosphorus compounds that found wide applications in catalysis, especially as bidentate ligands. Monophosphonites have thus been rather disregarded, in this respect, although they represent good substrates that can be diversely functionalized in order to tune their steric and electronic properties, affording effective catalysts for different processes. To evaluate this, a series of enantiomerically pure MOP-based structures have been prepared [30] (Figure 1), bearing –H or –OMe groups as the substituents on the 2′ position of the binaphthyl backbone, and phenyl or diphenyl groups on the phosphonate moiety to increase the bulkiness of the ligand. The relative cationic Rh(I) complexes were thoroughly studied for their coordination features, using both experimental and computational data to quantify their stereoelectronic donor properties. These complexes were tested in a series of asymmetric transformations, such as the hydrogenation of prochiral alkenes, the hydroformylation of styrene, and the addition of phenyl boronic acid to an isatin. The best results were achieved in the hydroformylation reaction, with high yields and regioselectivities for the branched isomer using low catalyst loadings.

**Scheme 2:**
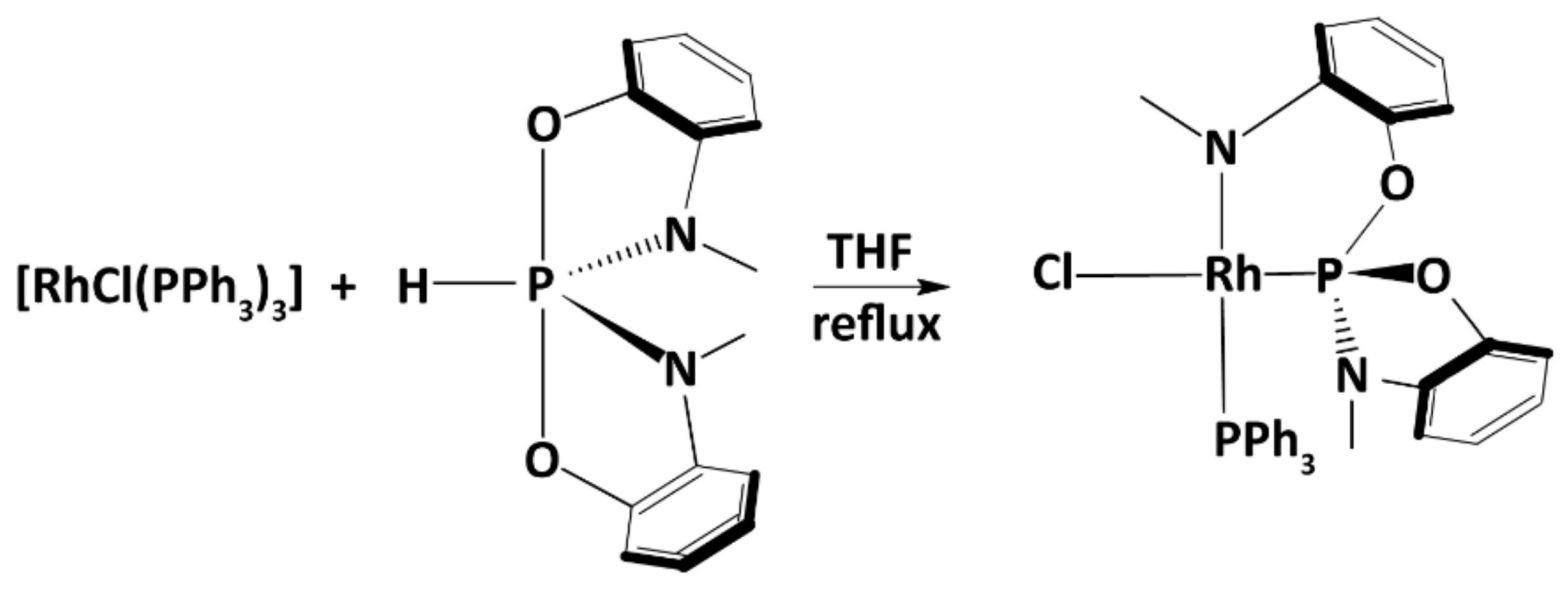
Reaction scheme for the synthesis of [RhCl(PPh_3_){P(OC_6_H_4_NMe)OC_6_H_4_NHMe}].

Phosphorus(V) species, such as hydrophosphoranes (HPR4), found their own niche in the chemistry of coordination compounds and catalysis [31] due to their unique binding modes and high activities. Some spirobicyclic hydrophosphoranes undergo ring-opening tautomerism, resulting in P(III) species. Ligand HP(OC_6_H_4_NMe)_2_, in its ring-opened form, was employed as a chelating phosphoramidite-amine ligand for the synthesis of a Rh(I) complex, [RhCl(PPh_3_){P(OC_6_H_4_NMe)OC_6_H_4_NHMe}], which resulted in a mixture of different diastereomers [32] (Figure 1, Scheme 2).

The catalyst was used in the hydrosilylation of 1-octene and 1-hexyne with various hydrosilanes, exhibiting good to high selectivities. A different kind of phosphoramidite ligand, based on the atropisomeric BINOL structure, was used in the preparation of a Rh(I) complex for the hydroformylation of 1-octene. The chiral compound displays the 1,2-diaminobenzene moiety as the backbone and two (R)-1,1′-binaphtalene-2,2′-dioxy (BINO) groups linked to the nitrogen atoms [33] (Figure 1). Thus, the structure of this ligand seems to be ideal for hydroformylation reactions, due to the presence of a flat and stiff skeleton bearing two bulky moieties (the binaphthyl groups) with a defined configuration. In fact, the Rh complex gave excellent aldehyde selectivities and n-regioselectivities under mild conditions, being the first catalyst of this kind to be so effective.

### 2.3. Immobilization and Heterogenization

**Scheme 3:**
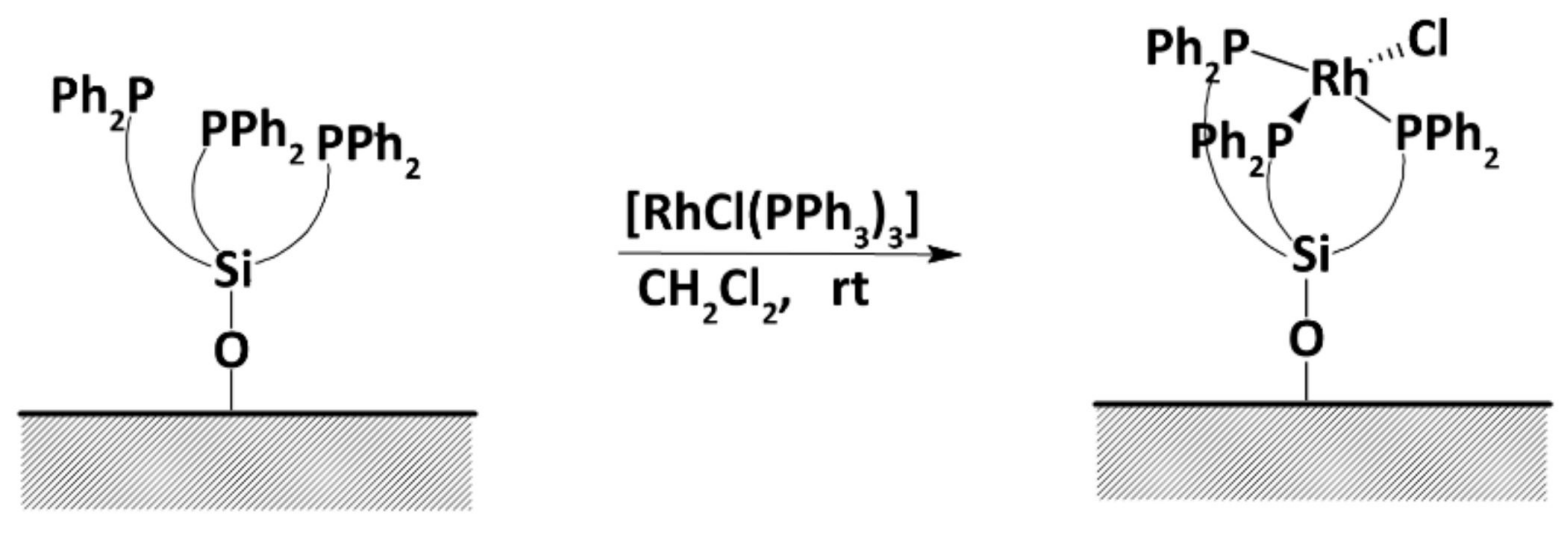
Reaction scheme for the synthesis of immobilized Rh-POL-PPh_3_.

Catalyst immobilization on solid supports is an important strategy to increase the sustainability of a chemical process, especially on the industrial scale, where large amounts of transition metal catalysts are involved. Such compounds are rather costly and may pose a serious threat to the environment in case of leaks and contaminations. Thus, catalyst separation and recycling are highly desirable in industrial transformations, and this led to the search for innovative phosphorus-based hybrid materials where immobilization on solid support was exploited to optimize such processes. Nanomaterials in this respect are gaining much attention, especially those based on nanocarbon structures or magnetic nanoparticles. The heterogenization of homogeneous metal complexes includes, for instance, the immobilization onto porous systems, such as POP (porous organic polymers) and the self-polymerized POL (porous organic ligands) possessing a high number of exposed phosphorus or nitrogen donors to block the metal centre or its ions in single sites. An example of this can be found in the Rh(I) complex with POL-PPh_3_ (Scheme 3), which was used in the heterogeneous carbonylation of methanol carried out in a continuous fixed-bed reactor, showing higher activity than that of the Monsanto process, which was maintained for nearly 180 h [34].

Another approach was the synthesis of rhodium complexes with some tripodal phosphine ligands incorporating long methylene chains of silicon-based compounds, such as silica or ethoxosylanes [35]. Finally, as nanomaterials are gaining growing attention also as good supports for metal complexes in catalytic processes, this strategy was applied for instance to the preparation of Rh complexes that were then appended onto phosphorus nanocarbons and magnetic nanoparticles with a covalent bonding, to avoid catalyst leaching. These new materials were used in the hydroformylation of styrene [36].

**Scheme 4:**
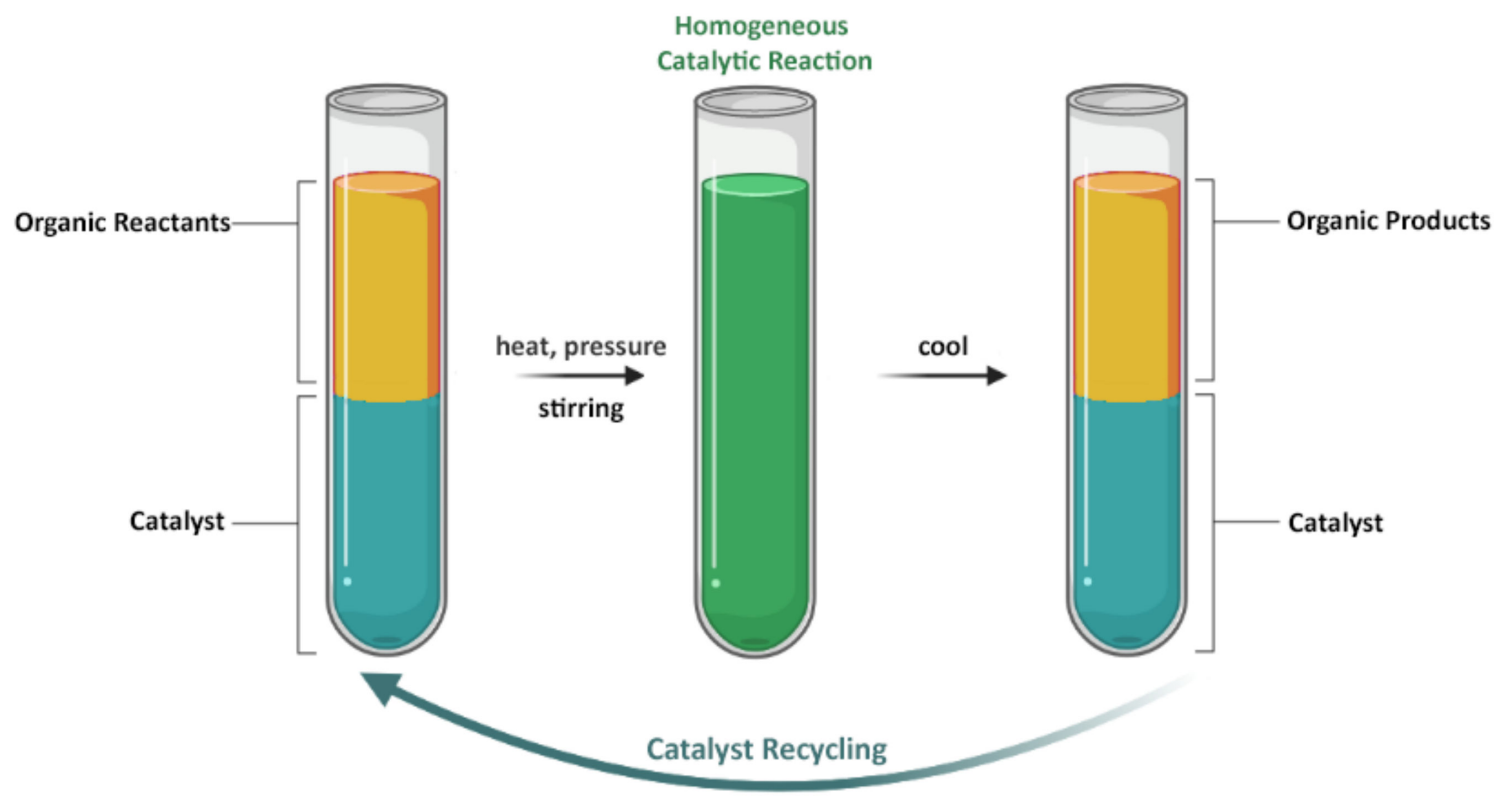
Description of a general biphasic process.

The possibility to carry out catalytic processes in biphasic systems, where the catalyst is immobilized in a different phase from the products and substrate, offers the opportunity of an effective recycling of the complex without having to append it onto a solid support. Aqueous and fluorous biphasic catalysis have been developed in this perspective. The latter is carried out in a system composed by the organic phase, consisting of a generic organic solvent, and the fluorous phase, consisting of a fluorinated solvent in which the fluorocarbon-containing catalyst is dissolved [37,38,39]. At room temperature, the two phases are immiscible, while by raising the temperature they turn into a single homogeneous mixture, allowing the catalyst and the substrate to react together. Once the temperature is lowered again, catalyst and product can be easily separated, each in a different phase (Scheme 4).

In this context, several fluorocarbon containing Schiff-base rhodium(I) complexes were prepared and applied to the hydroformylation of 1-octene carried out in a fluorous biphasic system [40] (Figure 1). A series of ligands have been synthesized as derivatives of triphenylphosphine, where one of the three aromatic rings was derivatized with an imine group to which a long fluorocarbon chain was appended. Other ligands were based on the salicylaldimine structure. All these species coordinated the rhodium centre in a heterobidentate fashion and showed their activity and selectivity toward aldehydes (major products) in the hydroformylation of 1-octene in a fluorous biphasic system.

**Scheme 5:**
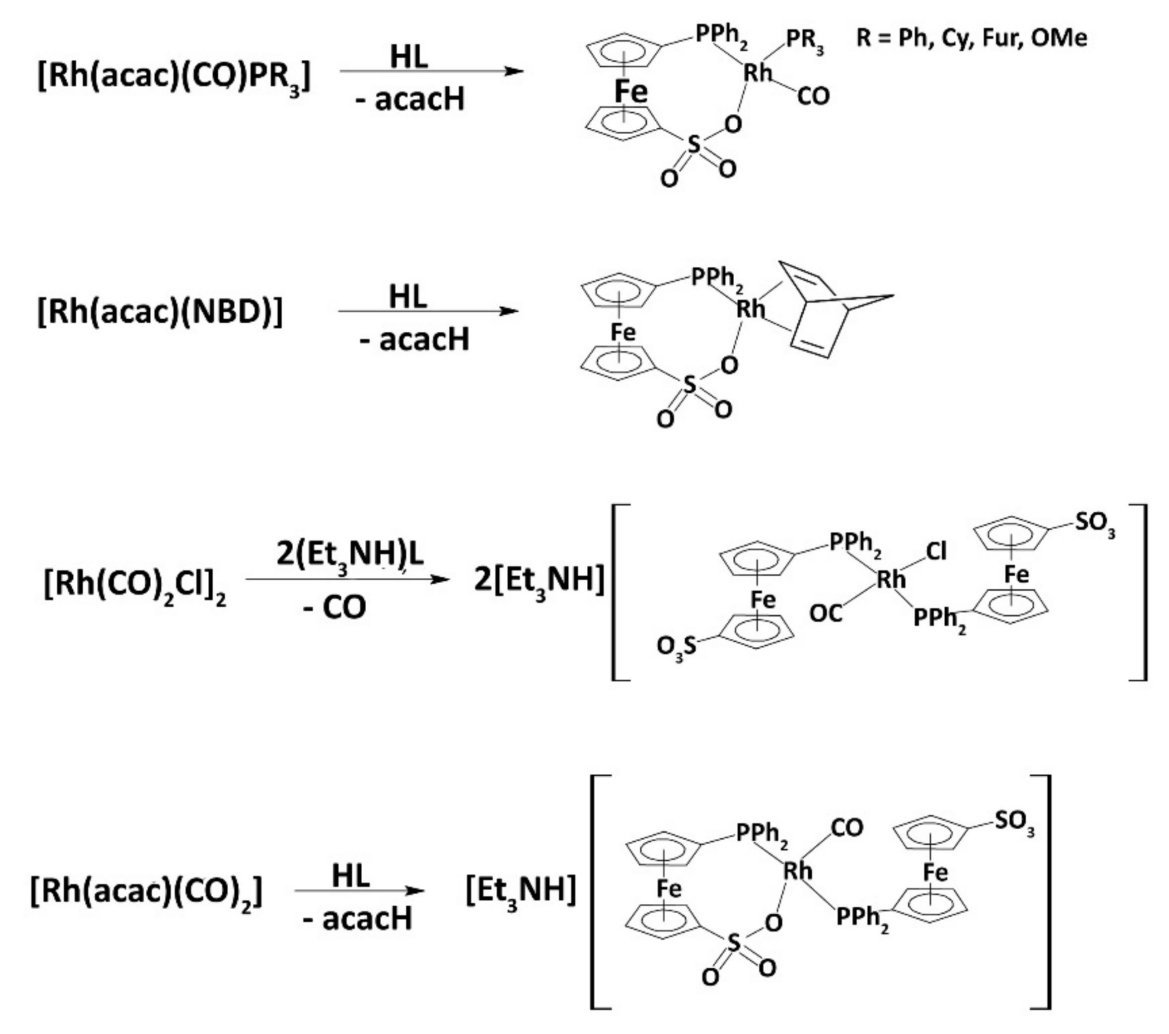
Reaction scheme for the synthesis of Rh(I) complexes with phosphinoferrocene amidosulfonate ligand.

Catalysis in aqueous phase, like the Ruhrchemie/Rhône-Poulenc process for propene hydroformylation (using the water-soluble Rh-TPPTS catalyst, TPPTS = triphenylphosphine-3,3′,3″-trisulfonic acid trisodium salt), is highly desirable, especially for industrial applications, since it decreases the use of organic solvents for a more sustainable chemistry. The sulfonic moiety seems indispensable to build a water-soluble complex, as it has been reported in a number of contributions [41,42,43,44,45], where the sulfonic group was successfully associated to phosphines in the design of new hydrophilic donors. During the past years, the synthesis of a series of novel Rh(I) complexes with phosphinoferrocene amidosulfonate ligands was reported, to be tested in the hydroformylation of n-hexene. These compounds, depending on the reaction conditions, can act either as monodentate or bidentate P,O-ligands, to give the corresponding Rh(I) complexes (Scheme 5, Figure 1) [46]. The reactions have been carried out under different compositions of the reaction mixture, going from solventless to water in the presence of toluene. The best conditions were achieved for water/toluene mixtures, where the catalysts are moderately active and selective in the hydroformylation reaction of alkenes (at 80 °C under 10 or 20 bar of synthesis gas), being limited by their dual nature: they are not completely soluble either in the neat alkenes used as the starting materials or in the solvents (water and biphasic aqueous mixtures).

## 3. N-Heterocyclic Carbene (NHC) Ligands 

NHCs are broadly employed in catalysis due to their structural plasticity, which guarantees the possibility of widely tuning their steric and electronic properties; moreover, the introduction of a donor group on the NHC backbone favours the formation of chelate complexes and thus represses ligand dissociation during the catalytic cycles. Several donor groups can be appended onto the imidazole nitrogen, to increase the complex stability. Such donor atoms can be directly bound to the nitrogen, or in some other cases a spacer can be interposed between them.

If phosphines are usually thought as the most effective ligands in catalysis, carbene complexes with most metals are more stable than those obtained with analogous phosphines, with similar or even higher performances in a number of processes [47]. The variety of structures for the NHC ligands is clearly evident from the reports in the literature [48], with a few new entries.

### 3.1. New NHC Ligands

**Figure 2:**
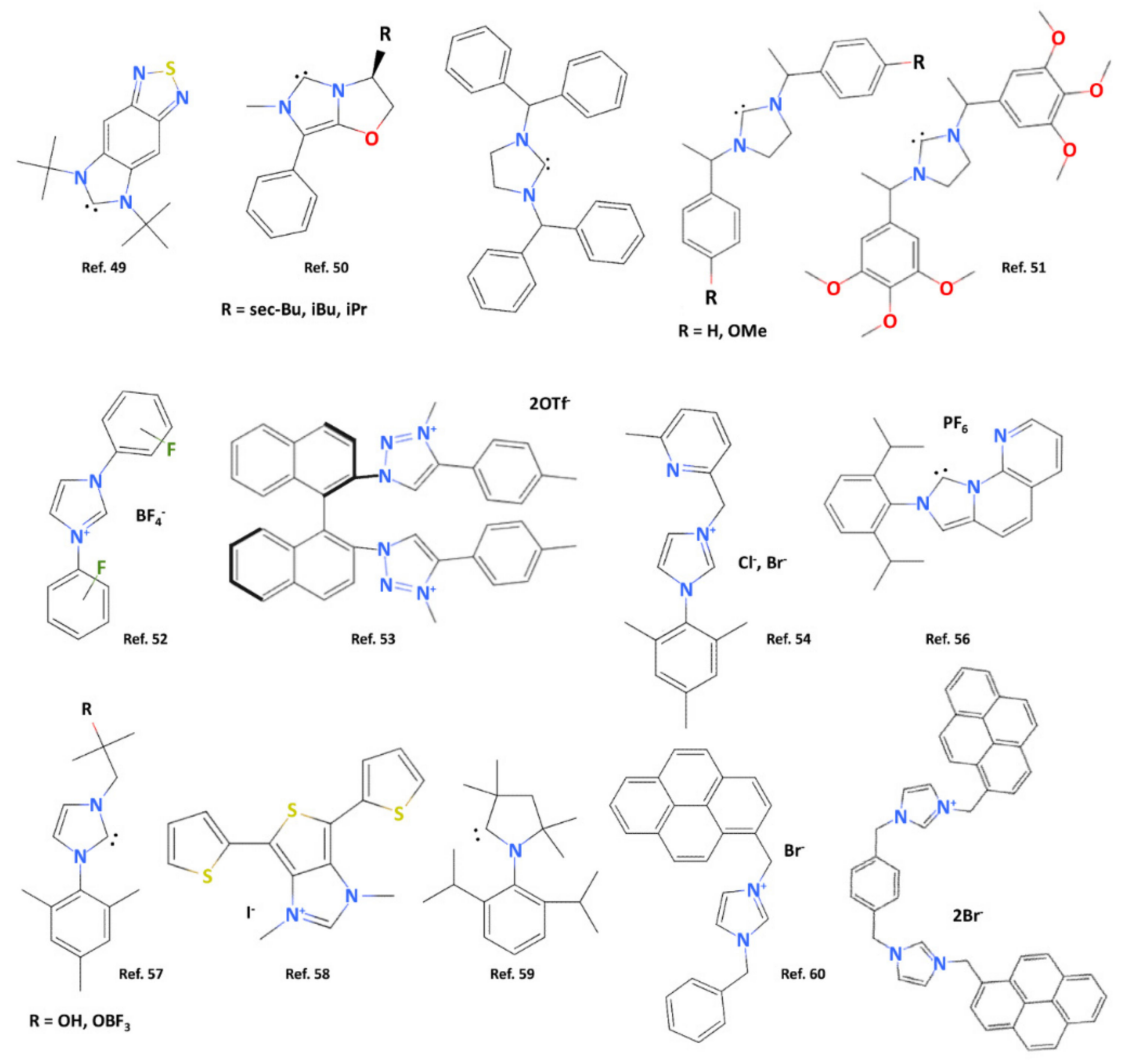
Molecular structures of some NHCs in recent literature.

A novel polycyclic NHC was synthesized, starting from a benzothiodiazole that was annulated onto the imidazol-2-ylidene structure to give a new ligand with the carbene centre as a part of a more extended, and aromatically rich, system, thus increasing its donor properties [49] (Figure 2). The relative Rh(I)(COD)L complex was also studied and preliminarily tested in the hydrosilylation of acetophenones, with interesting results.

Another type of new NHCs, bringing fused chiral oxazolidine rings, was prepared with the aim of combining the properties of these two classes of highly successful ligands in catalysis and improving their achievements in asymmetric synthesis. The chiral information, in fact, could be more easily transferred to the substrate thanks to its unique restricted rotation along the N–C bond bringing the chiral moiety, as it was demonstrated for the asymmetric transfer hydrogenation reactions of ketones [50] (Figure 2).

A different approach was applied to prepare 1,3-dialkylimidazolinium chloride salts starting from 1,2-bis(alkylamino)ethane and triethyl orthoformate, which were then used to obtain the relative Rh(I) complexes [51] (Figure 2). Their results in the hydrosilylation of acetophenone derivatives with triethylsilane showed an excellent catalytic activity with yields from about 80% to 96%.

Following a similar strategy, five N,N′-fluoroaryl NHC precursors were synthesized as their fluorinated imidazolium chloride salts by carrying out a one-pot condensation reaction using the proper fluoroaniline with glyoxal and para-formaldehyde [52] (Figure 2). Their rhodium complexes were tested in the hydrogen transfer reaction (hydrogenation of acetophenone with iso-propanol), with catalytic activities higher than those of the non-fluorinated NHCs counterparts, clearly showing the changes in activity due to the introduction of a fluorine substituent on the aromatic ring.

The series of novel ligands for Rh(I) catalyzed reactions includes a BINAM-based chiral di-1,2,3-triazolylidene, which was then deprotonated in the presence of a Rh(I) precursor to give the expected complex [53] (Figure 2). The aim of the study was to test the compound in the rhodium-catalyzed hydrosilylation reaction. This because NHC ligands possess strong electron-donating properties that favour their complexes in processes governed by the oxidative addition as the key (slow) step (e.g., hydrosilylation, allylic alkylation, and hydrogenation reactions). When the ligand is chiral, as in this case, it can be used in asymmetric catalysis on prochiral substrates. With this complex, the hydrosylilation reaction was performed on pro-chiral ketones, with good yields and catalyst loadings as low as 0.2 mol-%. However, the optical yield is still improvable.

### 3.2. Already Reported NHC Ligands

**Scheme 6:**
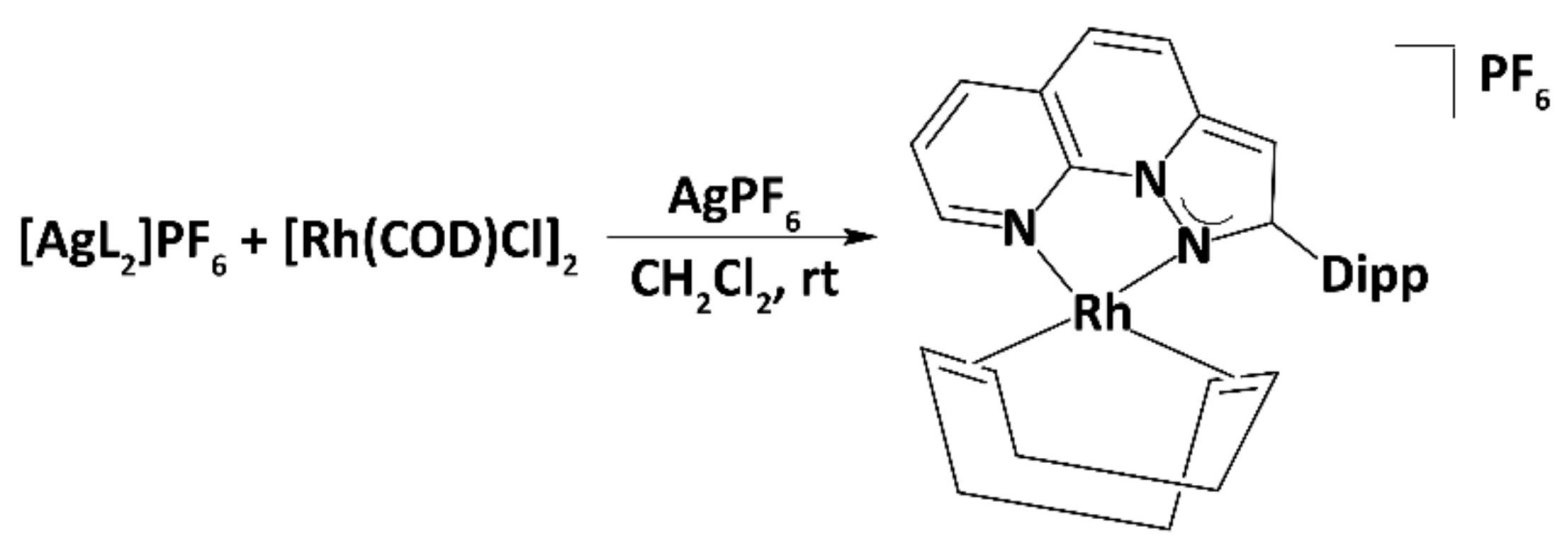
Reaction scheme for the synthesis of Rh(I) complexes with a fused π-conjugated imidazo[1,2-a][1,8]naphthyridine-based N-heterocyclic carbene ligand.

New complexes using previously reported ligands include a series of picolyl-NHC-containing Rh(I) species differing only in the steric hindrance at the 6-position of the hemilabile pyridine moiety. These complexes are able to reach high catalytic activities and E selectivities in the hydrosilylation of a vast gamut of terminal alkynes with different silanes [54] (Figure 2). Following this trend, an annulated NHC bearing a fused naphthyridine ring was prepared by a different research group, whose Rh(I) complex is analogous to that just mentioned and to others previously reported [55]. Additionally, this Rh(I) complex showed high performances in the E/Z selective alkyne hydrosylilation. The catalyst produced the E-vinylsylane in excellent yields and high selectivity [56] (Scheme 6, Figure 2).

Hydrosilylation and tandem isomerization–hydrosilylation of alkenes, on the other hand, were successfully performed by a family of rhodium complexes bearing an NHC ligand with an oxygen-containing pendant arm and differing for the derivatization on the O-donor [57] (Figure 2). The results showed the efficacy of O,C-hemilabile species in this kind of process, but also that the catalytic performances can be significantly affected by slight changes in the stereo-electronic properties of the ligand.

Terthiophene backboned N,N′-dimethylimidazolium species can be used as the precursors leading to Rh(I) complexes by in situ deprotonation, able to crystalize in two different enantiotropic packing polymorph phases. Both forms were catalytically active in the hydrogen transfer reaction of benzophenone with iso-propanol. Moreover, the two species can be converted to conducting metallopolymer thin films similarly to previously reported thiophene derivatives of functionalized salen [58] (Figure 2).

Cyclic (alkyl)(amino) carbenes (CAACs) differ from “classical” N-heterocyclic carbenes (bearing two nitrogen atoms adjacent to the carbene centre) in that they possess only one amino substituent, while the other was replaced by an sp^3^ carbon atom in an alkyl group. For this reason, CAACs are better σ-donors and π-acceptors with respect to “classical” NHCs. They also show higher nucleophilicity and electrophilicity, and were employed as ancillary ligands in rhodium-catalyzed arene hydrogenations of phenols and aromatic ketones, fluoroarenes, and silylarene. Rh(I) complexes of 1,3-bis(2,6-diisopropylphenyl)-1,3-dihydro-2H-imidazol-2-ylidene and 1,3-bis(2,4,6-trimethylphenyl)-1,3-dihydro-2H-imidazol-2-ylidene were prepared and used in the hydrogenation of (hetero)aryl boronate esters with retention of the boronate functional group, which can be further transformed into other products. This process allows the quick preparation of cis-substituted borylated cycloalkanes that are usually difficult to obtain by conventional synthetic strategies [59] (Figure 2).

### 3.3. Immobilization and Heterogenization

Finally, it has to be reported that Rh(I) complexes with NHCs were also immobilized on reduced graphene oxide to be tested in heterogeneous catalysis. This is the case of two pyrene derivatives of a classic imidaziole-based carbene: the asymmetric mono-NHC and its symmetric di-NHC derivative, where the two pyrene-NHC moieties are attached to the same phenyl ring. While the former is able to coordinate rhodium in a monodentate fashion, the latter is not capable of chelation, thus forming a bridged dinuclear species [60] (Figure 2). Both were grafted onto reduced graphene oxide and tested for their catalytic activity in the 1,4-addition of phenylboronic acid to cyclohexan-4-one and in the hydrosilylation of terminal alkynes. The dinuclear complex was always more active than the monometallic species in both the catalytic processes, and with the heterogenized catalysts, the trend was maintained. The solid containing the dinuclear catalyst was recycled five times without measurable loss of activity in the addition of phenylboronic acid to cyclohexanone. In the hydrosilylation of 1-hexyne, the use of the immobilized dinuclear species resulted in a neat increase of the selectivity of the transformation, giving the β-(Z)-vinylsilane with high stereoselectivity.

## 4. Cyclopentadienyl- and Ferrocenyl-Based Ligands

Cyclopentadienyl ligands are able to bind metals in a typical η^5^ fashion to give complexes that are widespread in organotransition metal chemistry because they are tightly bound to the metal centre and substantially inert towards substitution. Sandwich complexes containing one metal ion stuck between two cyclopentadienyl anions (Cp) are called metallocenes, some of which found application in olefin polymerization (early transition metallocenes). Such complexes often show redox proprieties: ferrocenium salts are oxidants, while cobaltocene is a strong reductant. Recently, group 9 cyclopentadienylmetal complexes of the CpM(III) type, where M is Co, Ir and especially Rh, were found to be catalytically active towards asymmetric C-H activation when bearing chiral Cp ligands [61]

**Figure 3:**
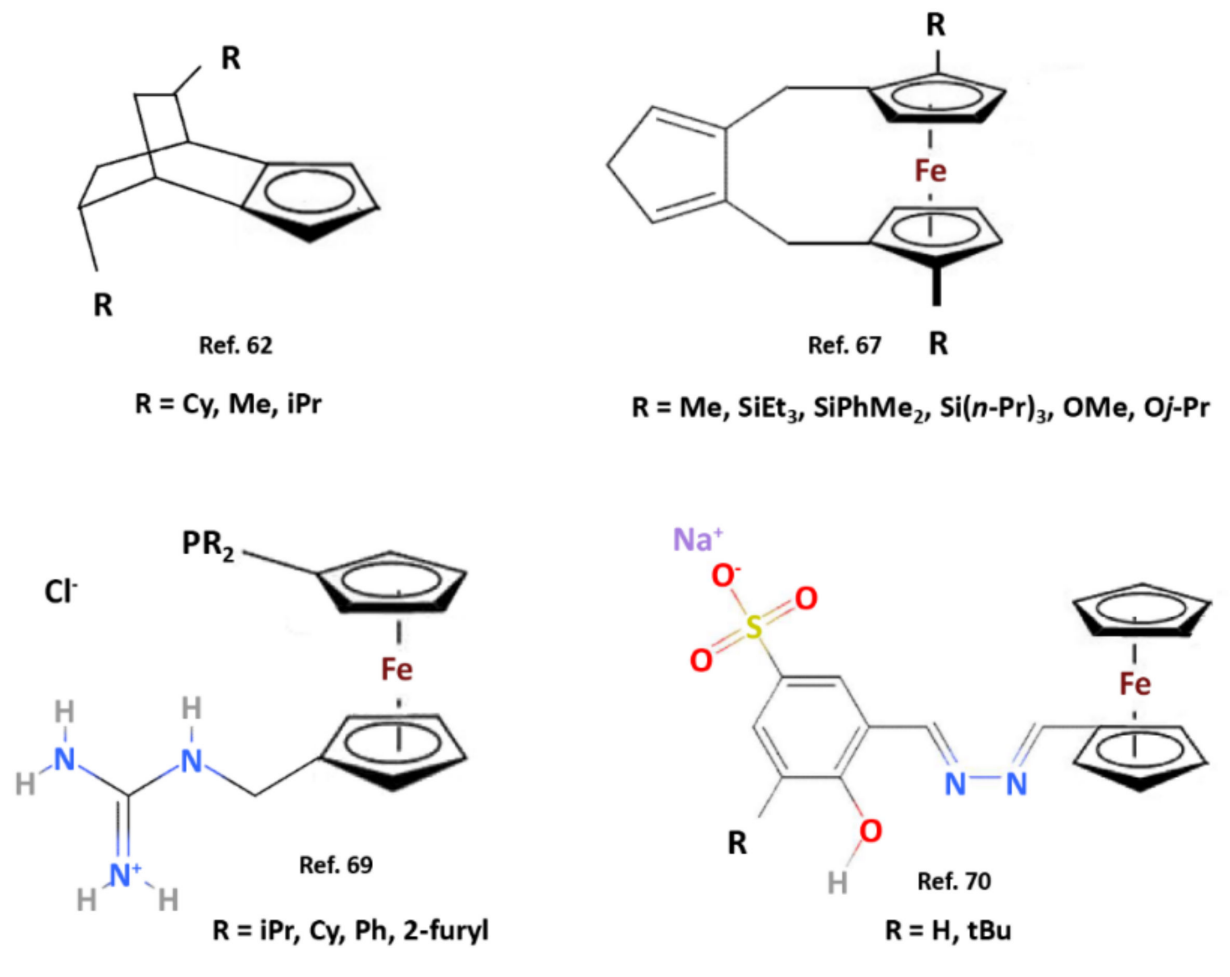
Molecular structures of some ferrocenyl-based ligands in recent literature.

Following this evidence, a series of chiral bridged-ring-based Cp catalysts were prepared and tested as their Rh(I) complexes in highly enantioselective C-H activation of quinones, with the aim of synthesizing important tricyclic hydrophenanthridinone intermediates. Different N-alkoxy benzamide substrates were tested with high yields and high enantioselectivities (up to 82% yields and 99% ees) [62] (Figure 3).

**Figure 4:**
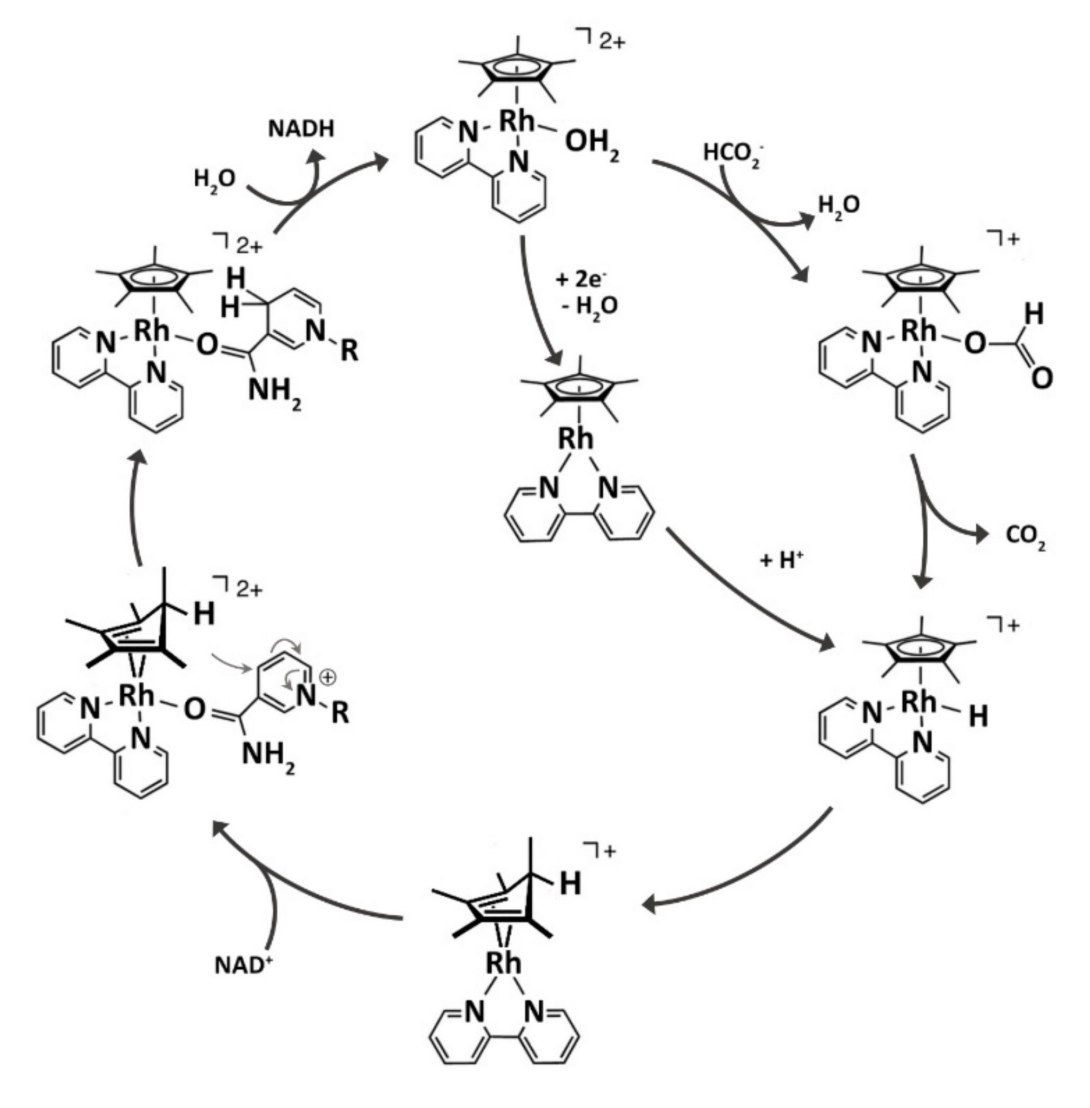
Proposed mechanism for NAD^+^ reduction with [Cp*Rh(bpy)]^+^ species.

1,2,3,4,5-pentamethylcyclopentadienyl ligand, briefly Cp* (or C_5_Me_5_), is a Cp derivative often exploited in the preparation of metal complexes with interesting catalytic properties. The steric bulkiness of the Cp* group allows the kinetic stabilization of otherwise highly reactive species. Moreover, the covalent bond between Cp* and the metal is relatively weak, enabling fast sigmatropic and haptotropic rearrangement processes, so that metal dissociation or ring-slippage can happen. In the case of a Cp*Rh(bpy)^+^ complex prepared for the chemical reduction of NAD^+^ to NADH, the reaction of the catalytic precursor with NaBH_4_ did not allow the isolation of the hydrido derivative, which further reacted to form an η^4^-pentamethylcyclopentadiene species [63]. This complex is a plausible intermediate in the Rh(I)-catalyzed reduction of NAD^+^ to NADH. Hydricity measurements and a series of H-transfer reactions to NAD^+^ and other transition metals confirmed this unexpected ligand-based hydride transfer reactivity, mediated by the otherwise innocent Cp* ligand (Figure 4).

An analogous behaviour was inferred for a Cp ligand derivatized with an alcoxysilane group, [C_5_Me_4_(n-propyl-Si(OEt)_3_)], which was used for the heterogenization of its relative R(I) complex. Immobilization of this compound on silica resulted in an effective catalyst for the olefin hydrogenation reaction, with a remarkable activity, high reusability, and limited rhodium leaching. ^13^C-CPMAS-NMR and DRIFTS deuteration experiments suggested a change in hapticity and the hypothesis that the hydrogenation reaction could proceed via a reversible intramolecular H-transfer process, from the metal centre to the cyclopentadienyl ligand [64].

Indenyl derivatives can be assimilated to Cp ligands, but their complexes show an increased catalytic activity with respect to cyclopentadienyl analogues due to what is known as the indenyl effect [65], the facile slippage of this ligand from an η^5^ to an η^3^ binding mode. Other advantages of indenyl derivatives are their structural flexibility and relative bulkiness, which favour ligand substitution and catalytic reactions. A series of arene rhodium complexes with the unsubstituted indenyl ligand were reported, with an aspect of novelty in the fact that this kind of coordination compounds are normally formed by the 1,2,3,4,7-pentamethylindenyl ligand only [66]. Their application in catalytic reductive amination of aldehydes produced the corresponding secondary and tertiary amines in very high yields (80−99%). The interesting feature of this process is that, among all solvents, it gives the best results in water.

However, the most used Cp-based backbone in the synthesis of Rh(I) complexes for catalytic reactions is indeed the redox active and chemically robust ferrocene scaffold (Figure 2), which gives heterobimetallic species where the active site is still the Rh(I) centre. An interesting series of novel C_2_-symmetric optically active Cp ligands incorporated the ferrocene scaffold in a ferrocene-bridged structure, which was made atropisomerically chiral via the 2,2′-subsitution with either methyl and silyl groups [67] (Figure 3). The corresponding Rh(I) complexes were tested in the asymmetric intramolecular amidoarylation of olefin-tethered benzamides via C-H activation, with very high yields and ees up to 67%.

The incorporation of a heterocyclic group at the α-ferrocenylmethyl position of the ferrocene scaffold represents one of the trends in the development of new ligands to be employed in catalytic processes for which classic ligands were not successful. To this framework, an extra donor can be added, especially phosphines. Such mixed donor species, of the P,N- or P,S-type, mostly in their chiral forms, have been employed in asymmetric processes, like hydrogenation, allylic alkylation, hydroboration, etc. A novel class of ferrocene/indole-based diphosphine ligands, named (Rc,Rp)-IndoFerroPhos, was prepared following these indications, where the heterocycle appended onto the α-ferrocenylmethyl position is the indole moiety, variously substituted at the N-atom. The main features of this new class of chiral diphosphines are their easy accessibility and facile derivatization, together with a remarkable air- and moisture-stability. The corresponding Rh(I) complexes achieved outstanding enantioselectivity in the asymmetric hydrogenation of functionalized olefins, with up to >99% yields and 98% ees [68].

**Scheme 7:**
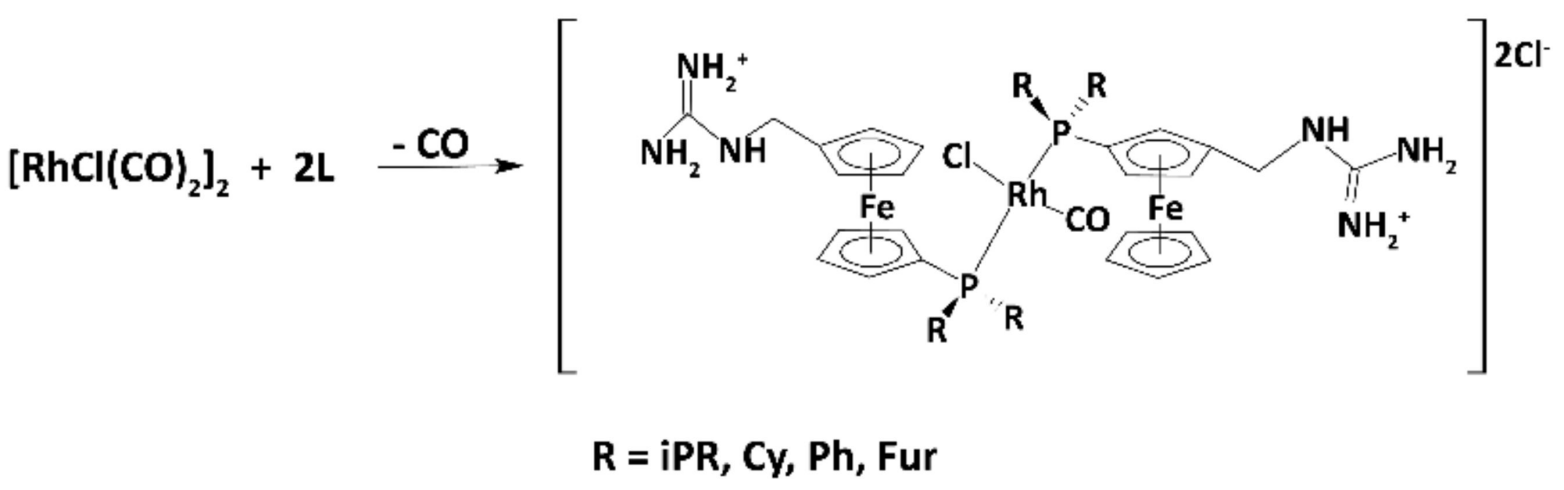
Reaction scheme for the synthesis of Rh(I) complexes bearing phosphanylferrocene ligands with cationic guanidinium tags and varied phosphane substituents.

Another example of phosphino-ferrocenes is given by the series of derivatives carrying a highly polar and positively charged guanidinium moiety onto the 1′-position, with the aim of increasing their affinity towards polar solvents. The hydrophobic phosphino group at the 1-position, on the other hand, was modified with both alkyl or aryl substituents [69] (Figure 3). The relative Rh(I) species, to which two ligands are bonded in a monodentate fashion (Scheme 7), were tested in the hydroformilation of 1-hexene, with yields going from moderate (42%) to high (98%) according to the substituent on the phosphorus atom.

The attempts to prepare water soluble complexes for biphasic catalysis find an example in the synthesis of a ferrocene substituted with sulfonato salicylaldimines to give mononuclear 5-sulfonatosalicylaldimine-ferrocenylimine Rh(I) complexes, which were then tested in the aqueous–organic biphasic hydroformylation of 1-octene, with good chemoselectivity towards aldehydes in the first cycles. The limit of these catalysts was shown by the poor conversions obtained after the fourth and fifth cycle, due to the catalyst leaching, with nearly a 90% loss of metal [70] (Figure 3).

## 5. Pincer Ligands

Pincer ligands are chelating species able to coordinate the metal in a tridentate way, so that they form three adjacent and coplanar rings around it (Figure 2). This rigid arrangement confers a high stability to the complex, which is further stabilized by the substituents on the donor atoms creating a hydrophobic pocket surrounding the metal ion. Classic pincers are those able to form cyclometalated complexes [71] such as the ECE (e.g., PCP, NCN, SCS, and PCN) [72] type, but PNN, PNO, PNP, and NNN compounds have also been proposed for catalytic purposes, such as the PNN ligand forming the so-called Milstein’s catalyst with ruthenium [73]. PCPs have probably been the most studied class of pincer ligands and extensively applied in catalysis, even with their P-chiral derivatives [74], but many other species have also gathered good success in this field due to their versatility and propensity to modification or derivatization, for a better tuning of the complex properties. Nevertheless, to date they have not found any industrial application.

**Figure 5:**
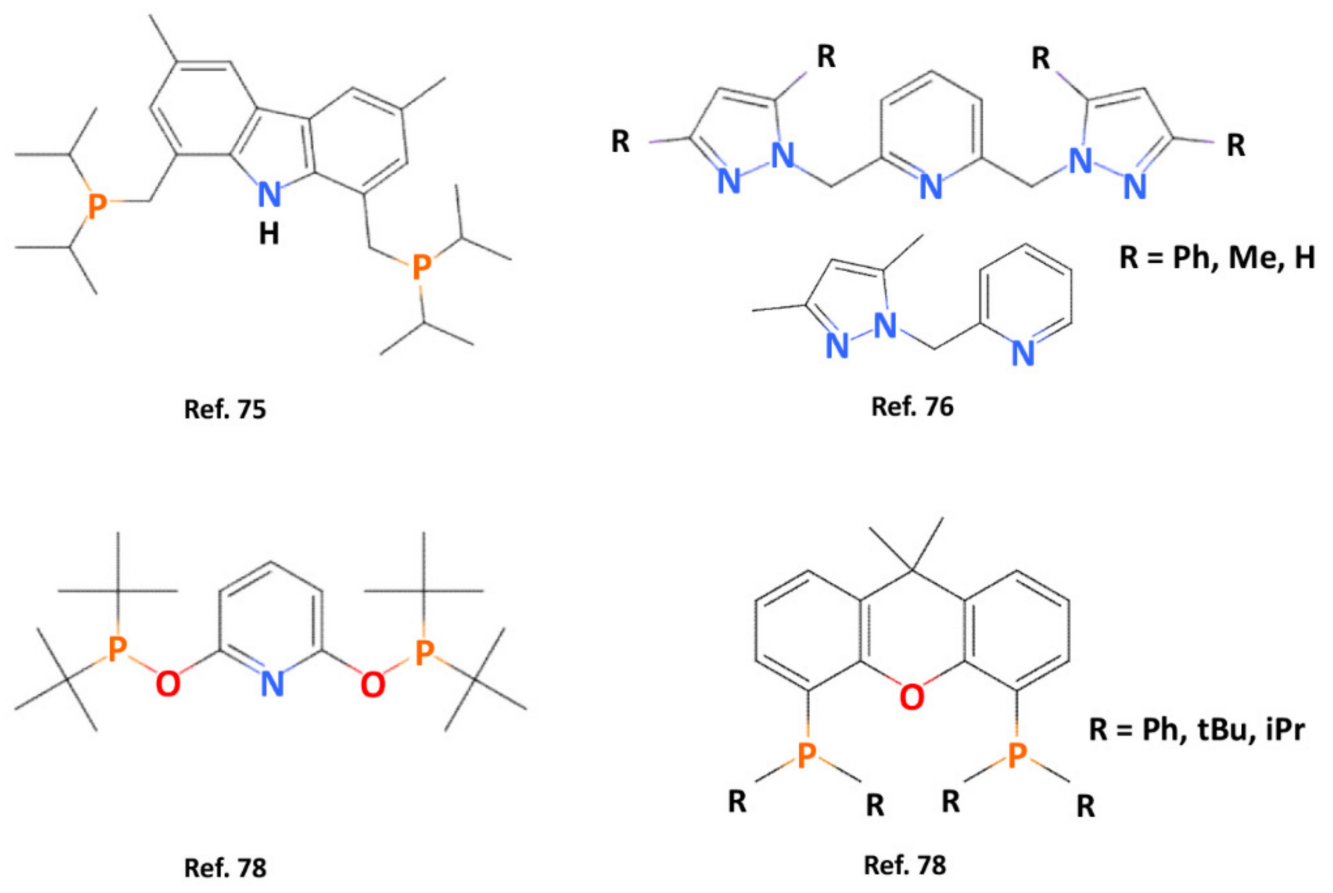
Molecular structures of some pincer ligands in recent literature.

During the last five-year period, only very few rhodium(I)-pincer complexes appeared in the literature for catalytic purposes. For instance, a previously reported carbazolide-based PNP pincer was used to prepare a rhodium(I) complex and tested in the alkane dehydrogenation reaction. Together with the experimental catalytic runs, a computational study was also carried out to elucidate the mechanism of this process and its rate-determining step [75] (Figure 5). The formation of an agostic intermediate complex was also evidenced.

A series of pyrazolylpyridyl N′N′N pincer ligands was prepared ex novo for the synthesis of the relative Rh(I) complexes and compared to an analogous bidentate pyrazolylpyridyl N′N rhodium species in the hydroformylation reaction. The parameters of this process were optimized by variating the syngas (CO and H_2_, 1:1 ratio) pressure, reaction time, and temperature, but also catalyst loading, to find the best reaction conditions. When 1-octene was the substrate, high conversions (up to 99%) and selectivities (up to 76%) were obtained (20 bar syngas pressure, 75 °C, over 16 h). Under these reaction conditions, both the electronic and steric properties of the complex are determinant: bulky and electron-withdrawing substituents were less successful in the hydroformylation, while donating methyl substituents or less bulky species but electron-donating methyl substituents were observed to be better-suited for the hydroformylation reaction when compared to the N′N bidentate complex [76] (Figure 5).

Neutral pincer PONOP was used to prepare two novel σ-amine-borane pincer complexes with formula [Rh(PONOP)(H_3_B·NMe_2_R)][BArF_4_], where R = Me or H. The rhodium precatalyst Rh(PONOP)(η^2^-H_2_)][BArF_4_], had already been reported [77]. These species were used to study in detail the dehydrocoupling of amine-boranes, H_3_B·NRR′H [78] (Figure 5), giving evidence to the general idea, among all the other findings, that a hydride transfer/reprotonation path starting from a σ-bound amine-borane is a possible mechanism for such dehydrogenation reaction when cationic catalysts are employed.

A non-classical pincer ligand of the POP kind is based on Xanthphos structure (Chart 3), a diphosphine characterized by an exceptionally wide bite angle (108°) [79]. This diphosphine has been tested in a number of catalytic reactions in which group 9 metals were involved, such as carbonylation, hydroamination, hydroacylation and hydroboration, activation of H_2_/alkenes, silylation, and amine−borane dehydrocoupling processes [79]. The relative [Rh(Xanthphos)][BArF_4_] system was evaluated in the C−S activation processes, by carefully studying its mer-[Rh(κ^3^-_P,O,P_-Xantphos)(η^2^-PhCCPh)][BArF_4_] derivative as a source of the active species, the [Rh(κ^3^_-P,O,P-_Xantphos)]^+^ fragment, that can be easily involved in ketone directed C−S and C−H activation reactions with aryl−ketosulfides.

## 6. Miscellaneous

Many other ligands reported during the last few years to prepare rhodium complexes for catalytic applications do not fall into broad representative categories, so they will be collected in this mixed session.

Natural amino acids are often used to prepare coordination compounds with both catalytic and biomedical applications. For instance, L-phenylalanine, L-valine, and L-proline were introduced as anionic ligands in Rh-COD compounds to give chiral complexes for the oxidative coupling reaction between phenylacetylene and benzaldehyde derivatives, producing seven types of chromones via C-H bond activation. Yields were variable, comprised between about 13–88%, depending on the electronic nature of the substituents on the benzaldehyde derivatives [80]. Moreover, it was found that when the amino acid was L-phenylalanine with its large aromatic moiety, it could easily dissociate from the metal centre to leave an empty active site, thus increasing the catalytic activity of the Rh complex.

**Figure 6:**
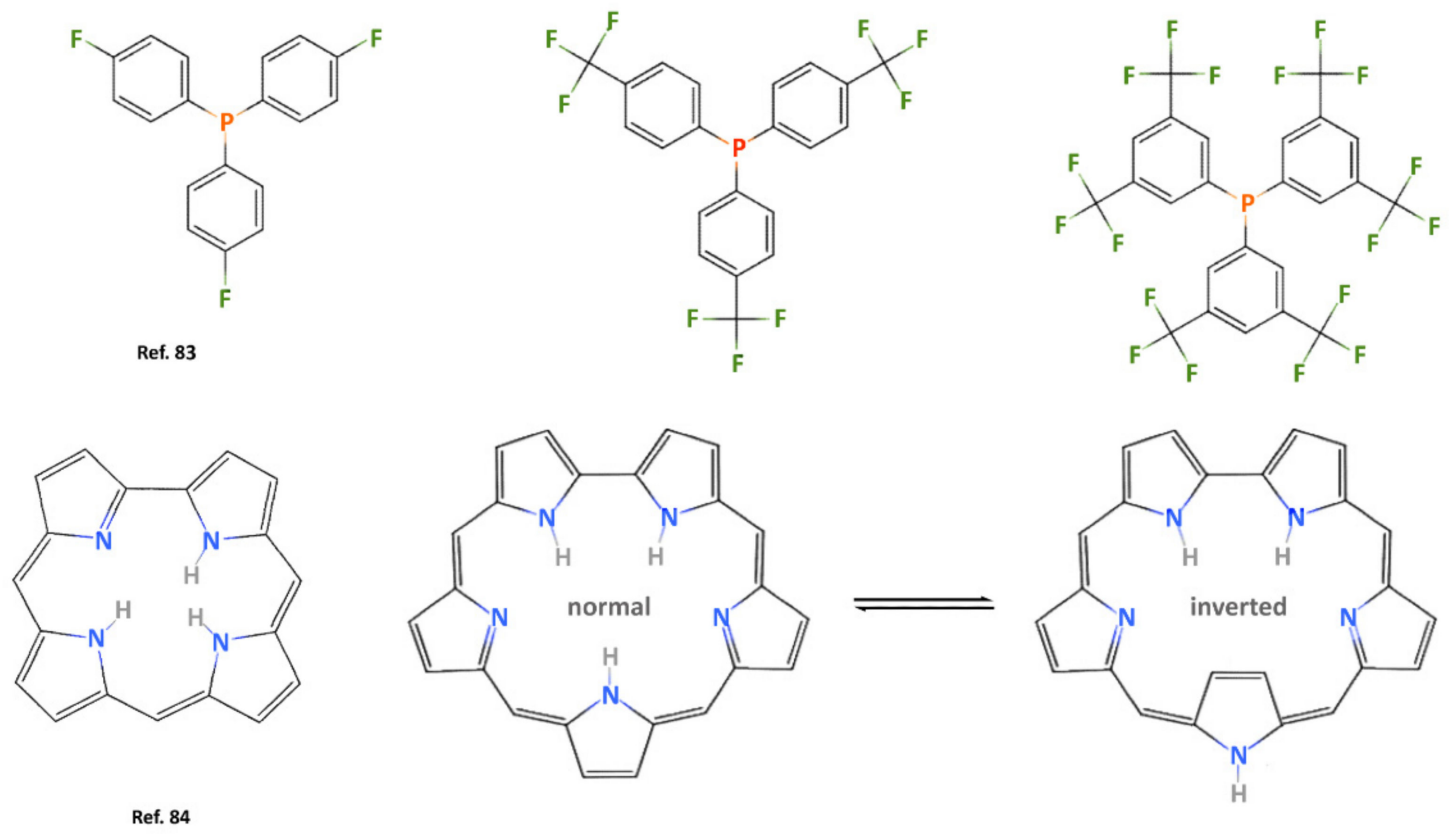
Molecular structures of some miscellaneous ligands.

**Scheme 8:**
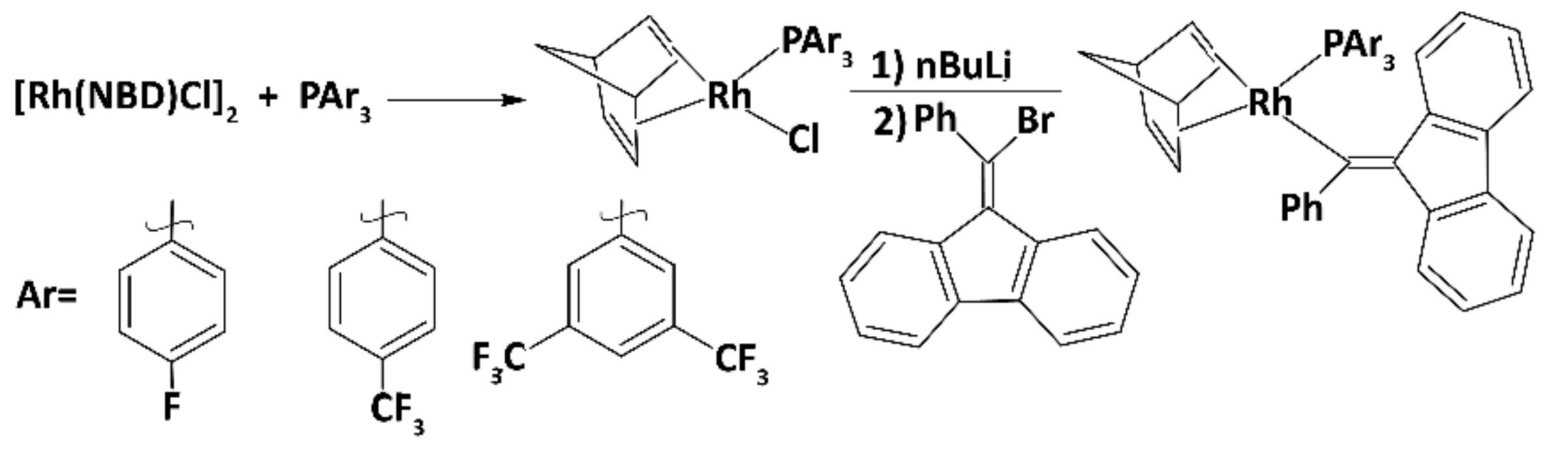
Reaction scheme for the synthesis of Rh(I) complexes containing the fluorenyl functionality in combination with fluorine functionalized phosphine ligands.

An analogous alaninate complex, [Rh(L-alaninate)(COD)], was synthesized, characterized, and applied to the asymmetric polymerizations of DoDHPA, a phenylacetylene substituted with a dodecyl and two hydroxyl groups. The substrate is achiral, but the resulting polymer has a favoured one-handed structure, induced by the amino acid chirality. Moreover, it has a higher molecular mass with respect to polymers obtained with [Rh(COD)Cl]_2_ as the initiator [81]. Comparable results were previously reported by the same research group with Rh(NBD)(l-proline) (NBD = 2,5-norbornadiene) on the same reaction [82], as [Rh(NBD)Cl]_2_ is another common initiator for this kind of process. An analogous idea was behind the synthesis of novel Rh(NBD) vinyl complexes (Scheme 8) containing a fluorenyl moiety and different fluorine-functionalized triarylphosphines for the homopolymerization of unsubstituted phenylacetylene [83] (Figure 6). Such complexes produced highly stereoregular polyphenylacetylene with *cis*-transoidal configuration (*cis* contents as high as 96%).

**Scheme 9:**
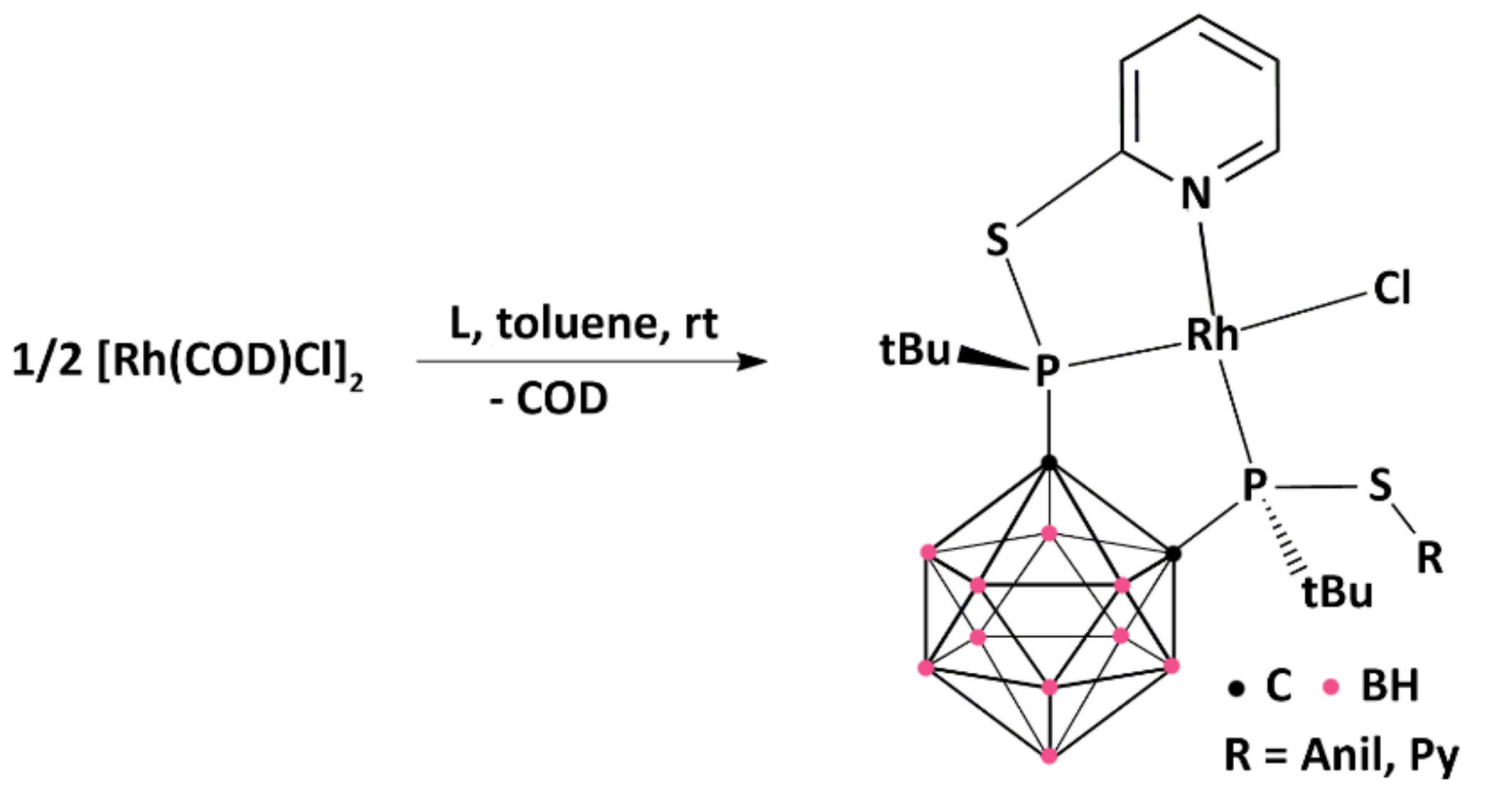
Reaction scheme for the synthesis of Rh(I) complexes with carborane-substituted P,N ligands.

To conclude this survey on the last five-year trends in the synthesis of Rh(I) complexes for catalytic applications, some interesting “exotic” structures that can be used as ligands for these coordination compounds should be cited, such as the sapphyrin or corrole derivatives and the substituted carboranes. In the first case, five new rhodium chelated complexes containing sapphyrins and corroles bearing fluorinated alkyl or aryl groups (C_6_F_5_ or CF_3_) on their meso-C atoms were prepared and characterized [84] (Figure 6), and their rich coordination chemistry was studied to understand the structural and electronic properties of these compounds, including elements of chirality. All the sapphyrine derivatives had one inverted pyrrole ring, and the crystal structure evidenced their C_1_-symmetric nature, leading to the corresponding enantiomers in a 1:1 ratio. A fast racemization of the two enantiomers was probably due to easy flipping of the metal centre through the core of the complex, but should be prevented by the use of bulkier co-ligands. The new compounds were found to be good catalysts in the cyclopropanation of styrene by ethyl diazoacetate. The meso-C_6_F_5_ derivatives were more selective towards cyclopropanation than dimerization of the substrate, with yields up to 85%, casting a light on the possibilities of catalytic applications of metallosapphyrins, which is a surprisingly unexplored field. In a similar way, icosahedral carborane structures have been rarely used as scaffolds for the synthesis of effective ligands in catalysis, although they possess unique and appreciable features from the point of view of the electronic properties and possibility of involvement of the B–H hydrogen in metal bonding. Carboranes can be conveniently derivatized to host, for instance, phosphine groups. Suitable further substitution with a “hard” donor such as nitrogen could afford hemilabile ligands, which are particularly useful in catalysis. The ortho-carborane backbone was thus exploited to build a series of P,N ligands, possibly also bearing a chiral centre [85]. The respective Rh(I) complexes (Scheme 9) were thoroughly studied to elucidate their properties and tested in the dehydrocoupling reactions of amine-boranes, and although they seem to be less active and selective than the reference complex [Rh(COD)Cl]_2_, their highly distorted square planar structure and the presence of hemilabile ligands could make them useful for other catalytic applications.

## 7. Conclusions

Despite the extensive exploration on “classical” ligands, such as phosphines, carried out during the past decades in the field of rhodium-catalyzed reactions, and the wide employment of other common types of ligands (e.g., NHCs, ferrocenes, Cp and Cp* derivatives, etc.), for this purpose, there is still lively research activity on this topic, with considerable efforts being made toward the synthesis of new preformed rhodium catalysts that can be both efficient and selective. Novel ligand structures have also been prepared and coordinated to this metal ion, with the aim of testing the new complexes in several processes, with very interesting and promising results. Although the “golden age” of homogeneous catalysis might seem over, there is still plenty of room for improvement, especially from the point of view of a more sustainable chemistry. The use of water-soluble TPA derivatives allows their employment in the aqueous medium, similarly to fluorine-substituted species that can be used in biphasic systems, while heterogenization with solid supports or nanomaterials is another emerging strategy to decrease the environmental impact of these catalysts and improve their recovery and reusability. From the examination of the most recent literature, it seems clear that rhodium-catalyzed processes still represent a stimulating challenge for the metalloorganic chemist that is far from being over.

## Abbreviations

Benzoxantphos	6,7-bis(diphenylphosphino)benzo[k,l]xanthene
Benzylnixantphos	10-benzyl-4,5-bis(diphenylphosphino)phenoxazine
BINAM	2,2′-bis(diphenylphosphinoamino)-1,1′-binaphthyl
BINAP	2,2′-bis(diphenylphosphino)-1,1′-binaphthyl
BINOL	1,1′-binaphthalene-2,2′-diol
BIPHEP	2,2′-bis(diphenylphosphino)biphenyl
BISBI	2,2′-bis(diphenylphosphinomethyl)-1,1′-biphenyl
Chiraphos	bis(diphenylphosphino)butane
COD	1,5-cyclooctadiene
Cy	Cyclohexyl
DPEphos	bis[(2-diphenylphosphino)phenyl] ether
DIPP	2,6-diisopropylphenyl
DPPB	1,4-bis(diphenylphosphino)butane
DPPE	1,2-bis(diphenylphosphino)ethane
DPPP	1,3-bis(diphenylphosphino)propane
DPPM	bis(diphenylphosphino)methane
DTBB	di-tert-butylphosphido-boratabenzene
Fur	Furane
iPr	Isopropyl
Isopropxantphos	4,5-bis(diphenylphosphino)-9-isopropylidenexanthene
Me	Methyl
MOP	2-(diphenylphosphino)-2-methoxy-1,1-binaphthyl
NBD	norbornadiene
Ph	Phenyl
PTA	1,3,5-triaza-7-phosphaadamantane
pTol	para-Toluene
SEGPHOS	5,5′-bis(diphenylphosphino)-4,4′-bi-1,3-benzodioxole
tBu	tert-Butyl
TPPTS	triphenylphosphine-3,3′,3″-trisulfonic acid trisodium salt
Nixantphos	4,5-bis(diphenylphosphino)phenoxazine
PCy_3_	tricyclohexylphosphine
Phosxantphos	4,5-bis(diphenylphosphino)-10-phenylphenoxaphosphine
DIOP	2,3-o-isopropylidene-2,3-dihydroxy-1,4-bis(diphenylphosphino)butane
DIPAMP	ethane-1,2-diylbis[(2-methoxyphenyl)phenylphosphane]
Duxantphos	4,5-bis[(2,5-dimethylphospholanyl)]-9,9-dimethylxanthene
Segphos	4,4′-bi-1,3-benzodioxole-5,5′-diylbis(diphenylphosphane)
Thixantphos	[7-(diphenylphosphanyl)phenoxathiin-4-yl]diphenylphosphane
Xantphos	4,5-bis(diphenylphosphino)-9,9-dimethylxanthene
